# Domestication of the green alga *Chlorella sorokiniana*: reduction of antenna size improves light-use efficiency in a photobioreactor

**DOI:** 10.1186/s13068-014-0157-z

**Published:** 2014-10-21

**Authors:** Stefano Cazzaniga, Luca Dall’Osto, Joanna Szaub, Luca Scibilia, Matteo Ballottari, Saul Purton, Roberto Bassi

**Affiliations:** Dipartimento di Biotecnologie, Università di Verona, Strada Le Grazie, Verona, 15-37134 Italy; Institute of Structural and Molecular Biology, University College London, London, WC1E 6BT UK

**Keywords:** *Chlorella sorokiniana*, Photosynthesis, Photobioreactor, Biomass, Biofuel, Antenna size, Light-use efficiency

## Abstract

**Background:**

The utilization of biomass from microalgae for biofuel production is one of the key elements for the development of a sustainable and secure energy supply. Among the different microalgae, *Chlorella* species are of interest because of their high productivity, high lipid content, and resistance to the high light conditions typical of photobioreactors. However, the economic feasibility of growing algae at an industrial scale is yet to be realized, in part because of biological constraints that limit biomass yield. A key issue is the inefficient use of light due to uneven light distribution, and the dissipation of excess absorbed light as heat. The successful implementation of biofuel production facilities requires the development of algal strains with enhanced light use efficiency in photobioreactors. Such domestication strategies include decreasing the absorption cross section in order to enhance light penetration, increasing the size of metabolic sinks per chlorophyll and minimizing feedback energy dissipation.

**Results:**

In this work we applied random mutagenesis and phenotypic selection to the thermotolerant, fast-growing *Chlorella* species, *C. sorokiniana*. Truncated antenna mutants (TAMs) were selected that exhibited a lower fluorescence yield than the wild-type (WT) strain. Six putatively interesting mutants were selected by high throughput fluorescence video imaging, two of which, TAM-2 and TAM-4, were found to have approximately half the chlorophyll content per cell and LHCII complement per PSII with respect to the WT. In batch culture, TAM-2 showed an increased photon use efficiency, yielding a higher P_max_ at saturating irradiances with respect to the WT. Cultivation of TAM-2 in both laboratory-scale and outdoor photobioreactors showed higher productivity than WT, with a 30% higher biomass yield in dense cell suspensions typical of industrial photobioreactors.

**Conclusions:**

These results suggest that generation of mutants with low chlorophyll content can significantly improve the light-to-biomass conversion efficiency of *C. sorokiniana* under mass culture conditions. However, owing to the lack of sexual reproduction in this species, the presence of additional mutations might affect growth rate, suggesting that selection should include evaluation of multiple independent mutants for each desired phenotype.

**Electronic supplementary material:**

The online version of this article (doi:10.1186/s13068-014-0157-z) contains supplementary material, which is available to authorized users.

## Background

In the last decades, the use of microalgae as a viable energy alternative to fossil fuels has attracted great attention, and efforts have been made to improve the mass culture yield using sunlight as the energy source. Microalgae have significant potential for biomass accumulation and biodiesel production when compared to crops, due to their higher productivity per surface and avoidance of competition for arable land for food production [1,2]. Due to their simple cellular structure, microalgae have a faster growth rate and are productive all year round; therefore, their biomass yield per area greatly exceeds that of the best crops [3]. Algae can grow in a broad range of temperature, salinity, and pH, and their ability to carry out photosynthesis under high CO_2_ concentrations [4] enhances the economic impact of algal-based technologies, due to their potential for CO_2_ mitigation [5].

Among the many candidate algal strains for biotechnological applications, a genus of considerable interest is *Chlorella*. Several freshwater species of *Chlorella* have been extensively used commercially over the past 40 years as a food and feed supplement on account of their rapid growth and tolerance over a wide range of temperature and culture conditions [6]. Cultures of *Chlorella vulgaris* grown in suitable outdoor photobioreactors (PBRs) can produce up to 40% of lipid per dry cell weight [7,8].

So far, algal-based industrial facilities have focused on the production of bioactive or dietary supplements due to their high product values [9], rather than on biofuels whose production is considered economically unfavorable [10]. An improvement in the biomass yield of microalgae in PBR conditions is therefore of primary importance and requires development of strains that overcome the biological constraints that limit intensive cultivation.

One of the most critical factors for biomass production is the efficiency of light utilization. Indeed, although photosynthesis has been optimized over three billion years of evolution, it remains inefficient at converting solar energy into chemical energy and biomass. The theoretical photoconversion efficiency of about 27% drops to 6% due to reductions in the efficiency of photon utilization and biomass accumulation [11]. Indeed, although the theoretical maximum productivity of microalgae is estimated to be around 170 to 190 g DW m^-2^ d^-1^ [11], the reported efficiencies in ponds or PBRs ranged from 20 to 35 g DW m^-2^ d^-1^ [12,13] with the present technology and available strains.

The reasons for this efficiency drop in dense culture conditions are rooted in the very structural organization of the photosynthetic apparatus. Oxygenic photosynthesis is performed by four multisubunit membrane-protein complexes in the thylakoid membrane: two photosystems (PSI and PSII), cytochrome *b*_6_*f*, and ATPase [14]. Each photosystem includes a core complex that binds cofactors involved in electron transport together with additional chlorophyll (Chl) *a* and β-carotene as antenna pigments. Associated with the photosystems is an array of antenna complexes called light harvesting complexes (LHCs) which bind Chl *a*, *b* and xanthophylls, and enhance photon absorption and transfer excitation energy for photochemical reactions [15,16]. LHCs also have essential roles in photoprotection, through the dissipation of excess light as heat (non-photochemical energy dissipation, NPQ), and in reactive oxygen species (ROS) scavenging [17-20]. A reduction in pigment content per cell and a reduction in antenna size are targets for optimizing the photosynthetic yield of unicellular algae under mass culture [21,22]. Indeed, the light use efficiency of microalgae in PBRs is limited by the steep light gradient due to the strong optical density of the near-molar concentration of Chls in cells. Microalgae have evolved in natural environments where light and inorganic elements, particularly iron, are often limiting, leading to a low cell density. As a survival strategy, large antennae were developed around photosystems in order to maximize their capacity to collect photons per unit of iron content [23]. Thus, cells in surface-exposed layers of PBRs absorb far more photons than they can use in electron transport and yet cell density and light intensity need to be high in order to increase productivity per unit of an installed facility [7]. Indeed, due to their huge optical density, the surface layers of microalgae easily reach saturation (and hence, photoinhibition) of photosynthesis [24], while the inner layers are light limited [25]. This non-homogeneous light penetration results in a low productivity of the system. Optimization of the light quality and intensity within the culture volume can alleviate these constraints. This can be done by selecting a mutant strain with a reduced pigment content per cell resulting either from a truncated antenna size or a lower overall density of photosynthetic units per cell [22,26]. The resulting decrease in optical density per biomass unit would mitigate the steepness of the light gradient typical of cultures of wild-type (WT) strains in ponds or PBRs, with cells located in surface layers absorbing less photons and those in inner layers receiving more light, altogether resulting in a higher rate of growth [27].

In this work we report the isolation of truncated antenna mutants of *Chlorella sorokiniana* following UV mutagenesis. This species was chosen for its very high growth rates [28] and tolerance to temperatures as high as 42°C [29], parameters that offer clear advantages for large-scale production in PBRs [22]. Our characterization of six potential mutants identified TAM-2 and TAM-4 as having a reduced antennae size and Chl content per cell. However, only TAM-2 showed an increased photon use efficiency, giving higher P_max_ at saturating irradiances with respect to the WT. Importantly, cultivation experiments in both laboratory-scale and outdoor PBRs consistently showed higher biomass productivity with TAM-2 (30% higher than WT) in dense suspensions of cells.

## Results

### Isolation of truncated antenna mutants (TAMs) of *Chlorella sorokiniana*

**Figure 1:**
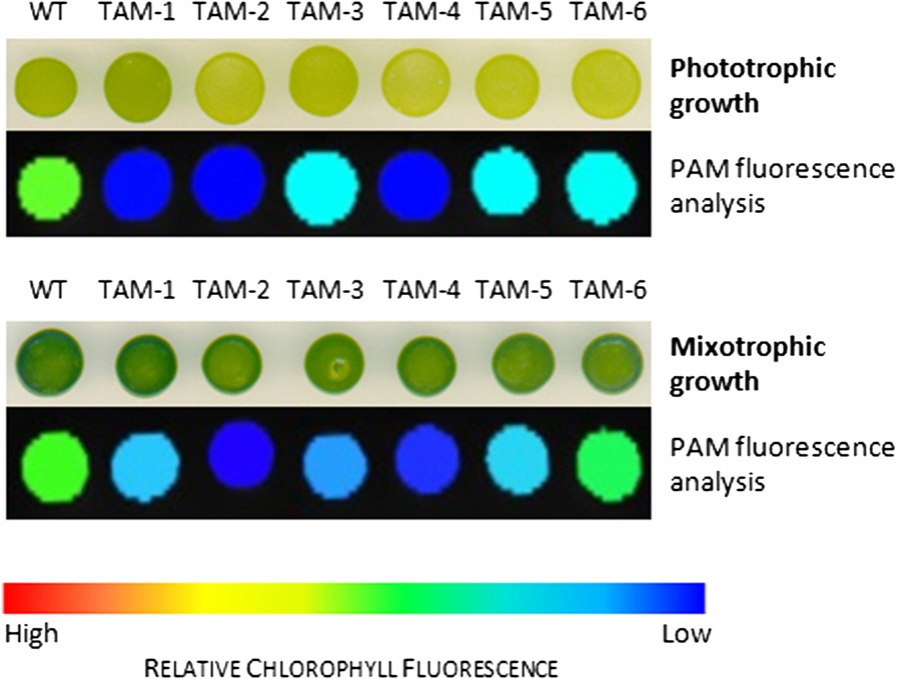
**Growth and fluorescence analysis of six putative truncated antenna mutants (TAM1-6) of** ***Chlorella sorokiniana*** **.** Culture samples were spotted onto minimal medium (upper panels: phototrophic growth) or acetate-containing medium (lower panels: mixotrophic growth), grown in continuous light (50 μmol photons m^-2^ s^-1^) for seven days, and then dark-adapted for pulse amplitude modulation (PAM) fluorescence analysis. False color images reveal that all six mutants have lower fluorescence emissions compared to the wild ype (WT), with TAM-2 and TAM-4 the most pronounced.

*C. sorokiniana* mutants that exhibited a lower Chl fluorescence yield than WT when exposed to a saturating pulse of light were identified following UV mutagenesis using a fluorescence video-imaging system (Additional file 1: Figure S1). Approximately 3,000 mutagenized lines were screened for retained photoautotrophy, but with a lower value of F_max_, which is expected to correlate with a smaller antenna size [16]. Six independent mutants were identified as putatively affected in antenna size (**t**runcated **a**ntenna **m**utants) and named TAM-1 to TAM-6. As shown in Figure 1, all six mutants are capable of phototrophic growth, but they display various levels of reduction in fluorescence, with TAM-2 and TAM-4 the most pronounced.

The pigment composition of the mutants and the WT strain were determined after five days of growth, as shown in Additional file 1: Table S1. Two of the mutants (TAM-2 and TAM-4) showed a significant reduction of Chl content per cell when grown in minimal medium, while the other mutants had a Chl content per cell similar to that of WT. Furthermore, the analysis showed that the Chl *a/b* ratio was significantly higher in TAM-2 and TAM-4 with values of 3.36 and 3.40, respectively versus 2.62 in WT, while the Chl/Car ratio was significantly lower in TAM-1, TAM-2, and TAM-4 with respect to the WT. These data suggest a depletion in the Chl *b*-rich light-harvesting antenna complexes in TAM-2 and TAM-4, and so these mutants were chosen for further study. Additional HPLC analysis of the carotenoid composition of DMFA-acetone cell extracts (Additional file 1: Table S2) revealed that TAM-2 and TAM-4 accumulate lower levels of neoxanthin and lutein on a Chl basis compared to WT, while both β-carotene and xanthophyll cycle pigments (violaxanthin, antheraxanthin, and zeaxanthin) were more abundant.

### Organization and stoichiometry of pigment-protein complexes

**Figure 2:**
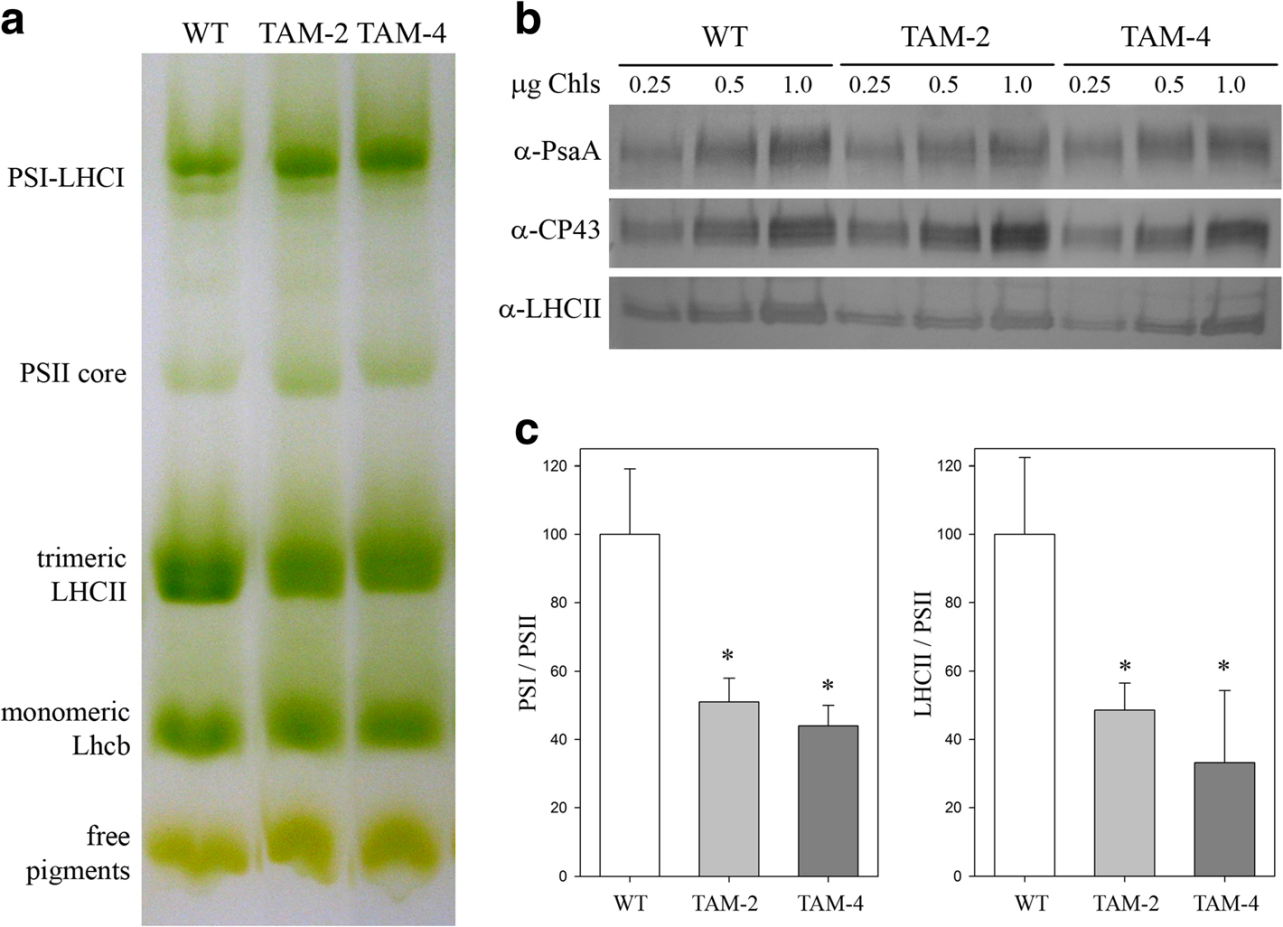
**Polypeptide composition of thylakoid membranes from wild-type, TAM-2, and TAM-4 mutants. (a)** Thylakoid pigment-protein complexes were separated by nondenaturing Deriphat PAGE upon solubilization with 1% β-DM. Thylakoids corresponding to 25 mg of chlorophylls were loaded in each lane. The composition of each band is indicated. **(b)** Immunoblotting used for the quantification of photosynthetic subunits in the wild-type (WT) and TAM thylakoids. Immunoblot analysis was performed with antibodies directed against individual gene products: LHCII, the major light harvesting complex of PSII; the PSII core subunit PsbC (CP43); the PSI core subunit (PsaA). Thylakoids corresponding to 0.25, 0.5, and 1 μg of Chls were loaded for each sample. All samples were loaded on the same SDS-PAGE slab gel. **(c)** Results of the immunotitration of thylakoid proteins. Data of PSII antenna subunits (left panel) and PSI core subunit (right panel) were normalized to the PSII core amount (PsbB content) and expressed as a percentage of the corresponding wild-type content ± SD. Significantly different values from wild type are marked with an asterisk.

Pigment-protein complexes from WT, TAM-2, and TAM-4 were separated by non-denaturing Deriphat-PAGE following solubilization of thylakoid membranes with dodecyl-β-D-maltoside. All three strains showed similar electrophoretic profiles with five major green bands resolved, as shown in Figure 2a. However, densitometric analysis of the Deriphat-PAGE showed differences in the TAM-2 and TAM-4 profiles with respect to the WT: namely a reduced level of trimeric LHCII and a higher relative abundance of PSII core complexes in both mutants. The level of selected thylakoid proteins was determined by immunotitration and expressed relative to WT on a Chl basis (Figure 2b): the LHCII content was reduced in both TAM mutants, amounting to about 48% of WT values in TAM-2 and about 35% in TAM-4. Moreover, the PSI/PSII ratio was reduced in both mutants, reaching approximately 50% with respect to the WT level (Figure 2c).

**Figure 3:**
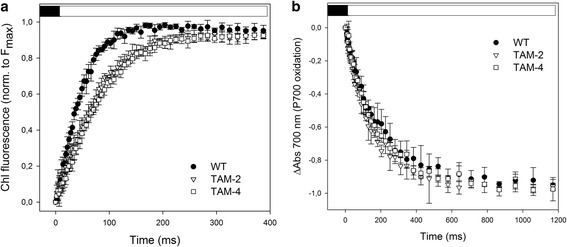
**Functional antenna size of PSII and PSI measured in wild-type and mutants TAM-2 and TAM-4. (a)** Variable Chl fluorescence was induced with a green light of 15 μmol photons m^-2^ s^-1^, on dark-adapted cells (about 1.0 · 10^7^ cells/ml) in BG-11 medium supplemented with 50 μM DCMU. The reciprocal of time corresponding to two-thirds of the fluorescence rise (T_2/3_) was taken as a measure of the PSII functional antenna size. **(b)** The kinetics of P700 oxidation (ΔAbs at 705 nm) were measured on thylakoid suspension (75 μg Chl/ml) treated with 50 μM 2,5-dibromo-3-methyl-6-isopropylbenzoquinone (DBMIB) and 1 mM methylviologen, upon illumination with a 10-s pulse of red actinic light (λ =630 nm, 560 μmol photons m^-2^ s^-1^). Data are expressed as mean ± SD, n =7.

The biochemical results were further confirmed through antenna size estimation for both photosystems. The PSII light harvesting cross section was measured by *in vivo* Chl fluorescence induction on cell suspensions in the presence of 3-(3,4-dichlorophenyl)-1,1-dimethylurea, (DCMU) (Figure 3a, Additional file 1: Figure S2). The T_2/3_ of the Chl fluorescence rise is inversely related to the functional antenna size of PSII [30] and was reduced by about 45% in both TAM-2 and TAM-4 mutants with respect to WT (Table 1, Additional file 1: Table S1). For PSI, the estimation of the functional antenna size was carried out by light-induced P700 absorption changes at 705 nm on thylakoid suspensions (Figure 3b). The antenna size was expressed by the T_1⁄2_ value, namely the time needed to oxidize 50% of the P700 in the sample. Results (Table 1, Additional file 1: Figure S2) show that there was no significant difference in T_1⁄2_ in the WT and TAM samples, suggesting that the PSI antenna size was unaffected by the reduction of Chl content per cell in the mutants.

**Table 1:** **Pigment content, maximum quantum yield of PSII, and functional antenna size of wild-type and mutants TAM-2 and TAM-4**

**Genotype**	**Chl/cell (pg)**	**Chl** ***a/b***	**Chl/Car**	**F**_**v**_ **/F**_**m**_	**PSII antenna size (T**_**2/3**_^**−1**^ **· 10** ^**3**^ **, ms-** ^**1**^ **)**	**PSI antenna size (T**_**1/2**_ **, ms)**
WT	0.49 ± 0.07^a^	2.62 ± 0.02^a^	3.43 ± 0.02^a^	0.69 ± 0.02^a^	18.2 ± 1.4^a^	134.3 ± 26.8^a^
TAM-2	0.30 ± 0.02^b^	3.36 ± 0.03^b^	3.07 ± 0.05^b^	0.70 ± 0.01^a^	10.5 ± 0.5^b^	115.4 ± 23.5^a^
TAM-4	0.34 ± 0.05^b^	3.41 ± 0.03^b^	3.18 ± 0.03^b^	0.69 ± 0.03^a^	9.4 ± 0.5^b^	122.7 ± 14.4^a^

Data are expressed as mean ± SD. Significantly different values (ANOVA, *P* <0.05) with respect to the wild-type (WT), within the same column, are marked with different letters.

### Light-saturation curve of photosynthesis

**Figure 4:**
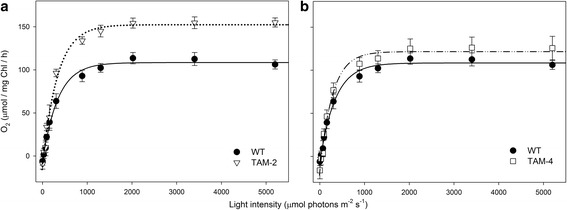
**Light-saturation curves of photosynthesis.** Curves were obtained with the *C. sorokiniana* wild-type and the TAM-2 **(a)** and TAM-4 **(b)** mutants. The light-saturated P_max_ was 1.5-fold higher in the TAM-2 mutant than in the wild type, suggesting a greater productivity on a per-Chl basis. In the TAM-4 mutant, the light-saturated O_2_ yield was not significantly higher than that for the wild type. Data are expressed as mean ± SD, n =4.

To investigate the functional properties of the photosynthetic machinery in the TAM mutants with respect to that in the WT, the efficiency of photosynthetic electron transport was measured for photoautotrophically grown cells. The maximal quantum yield of photosynthesis was found to be the same for the WT and TAM strains (Table 1), thus indicating no limitations in PSII charge separation. The light-saturation curve of photosynthesis was also measured in photoautotrophically grown cells (Figure 4, Additional file 1: Figure S3). The rates of O_2_ release were shown to increase as a function of irradiance within the range of light intensities of 0 to 1,200 μmol photons m^-2^ s^-1^. The increase was linear for WT and TAM mutants at irradiances below 200 μmol photons m^-2^ s^-1^. The slope of the linear regressions of O_2_ yield versus light intensity was 0.34 ± 0.04 and 0.39 ± 0.03 for TAM-2 and TAM-4, respectively, versus 0.28 ± 0.02 for WT cells, thus showing that the quantum yield of photosynthesis for both TAM mutants was significantly higher with respect to the WT. Moreover, this means that truncated antenna size mutants are not affected in the quantum yield of the photosynthetic apparatus. The intensity at which photosynthesis was half-saturated was similar in all the strains tested, at approximately 250 μmol photons m^-2^ s^-1^ (Table 2), and irradiances higher than 1,100 μmol photons m^-2^ s^-1^ did not lead to any further increase in the O_2_ yield in either WT or TAM mutants (Figure 4). It is worth noting that no decrease in O_2_ production was observed at very high light conditions (>3,000 μmol photons m^-2^ s^-1^), suggesting that these strains are rather resistant to photo-oxidative stress.

**Table 2:** **Photosynthesis and respiration rates**

**Parameters**	**WT**	**TAM-2**	**TAM-4**
Half-saturation intensity (μmol photons m^-2^ s^-1^)	275 ± 48^a^	259 ± 42^a^	238 ± 51^a^
P_max_ (μmol O_2_ mg Chl^-1^ h^-1^)	119 ± 7.2^a^	164 ± 7.3^b^	141.5 ± 12.0^a^
Respiration (μmol O_2_ mg Chl^-1^ h^-1^)	6.2 ± 3.0^a^	10.4 ± 4.4^a,b^	16.4 ± 4.3^b^
Respiration (fmol oxygen cell^-1^ h^-1^)	3.1 ± 1.5^a,b^	2.6 ± 1.2^a^	5.6 ± 1.5^b^
P_max_/respiration (relative units)	19.3 ± 9.4^a^	15.8 ± 6.7^a,b^	8.6 ± 2.4^b^

Parameters were measured on a dark-adapted cell suspension of wild-type (WT), TAM-2, and TAM-4, at seven days of photoautotrophic growth in BG-11 medium in low light conditions (70 μmol photons m^-2^ s^-1^, 25°C). O_2_ evolution/consumption were measured with a Clark-type oxygen electrode. Data are expressed as mean ± SD (n >4). Significantly different values (ANOVA, *P* <0.05) with respect to the WT, within the same row, are marked with different letters.

The values of P_max_, namely the maximum rate of light-induced oxygen evolution (photosynthesis net respiration) was measured at 2,000 μmol photons m^-2^ s^-1^ and was equal to 119 ± 7 μmol O_2_ per mg Chl per h in the WT, 165 ± 7 in TAM-2, and 141 ± 12 in TAM-4 cells (Figure 4 and Table 2). Since the rate of O_2_ production is normalized on the Chl content of the samples, the value of P_max_ is a measure of the productivity of Chl in the two strains. Interestingly, TAM-2 showed a significantly higher P_max_ than the WT (Table 2).

The dark respiration of the strains was measured and O_2_ consumption normalized to the Chl content of the cells: the respiration rates were 6.2 ± 3.0 μmol O_2_ per mg Chl per h in the WT, 10.4 ± 4.4 in TAM-2, and 16.4 ± 4.3 in TAM-4 (Table 2); when normalized to a per-cell basis, TAM-2 cells showed a lower (-16%) and TAM-4 a higher (+58%) oxygen consumption with respect to the WT. Consistent with the evidence of a higher respiration rate in the TAM-4 mutant was the value of light-saturated O_2_ yield of this strain, which was not significantly higher than that for the WT (125 ± 11 μmol O_2_ per mg Chl per h for TAM-4, compared with 114 ± 7 μmol O_2_ per mg Chl per h for the WT, see Figure 4b). TAM-2 showed a 40% higher O_2_ yield than the WT (Figure 4a).

### Cultivation of WT and TAM strains in photobioreactors

The results presented above indicate that the mutant TAM-2 is likely to have enhanced efficiency of light energy conversion and photosynthetic productivity with respect to the WT strain. Therefore, TAM-2 was chosen for detailed analyses of growth rate and biomass productivity. Photoautotrophic growth was monitored over a period of 10 days in a laboratory-scale PBR, a semi-batch cultivation system composed of 1-L glass cylinders illuminated by white light diodes at a light intensity of 450 μmol photons m^-2^ s^-1^. Cells were cultivated in minimal medium under a day:night cycle of 16:8 h. The system was fed with a flux of air and CO_2_, whose relative abundance was regulated by the pH of the medium in order to keep within the range of 6.8 to 7.1.

**Figure 5:**
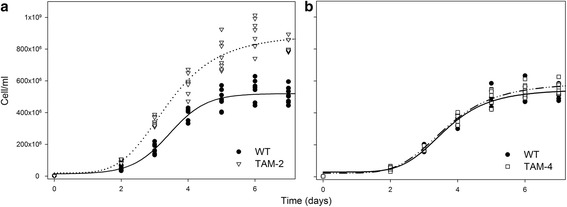
**Growth curves of wild-type, TAM-2, and TAM-4 mutants under autotrophic conditions.** Wild-type was grown with TAM-2 **(a)** or TAM-4 **(b)** and the cell content was measured once a day. All experiments were performed in 1-L cylinders, illuminated with 450 μmol photons m^-2^ s^-1^, 25°C. Growths were performed in a semi-batch system fed with air/CO_2_ mix; the CO_2_ supply was modulated in order to keep the pH of the medium always below 7.1.

**Figure 6:**
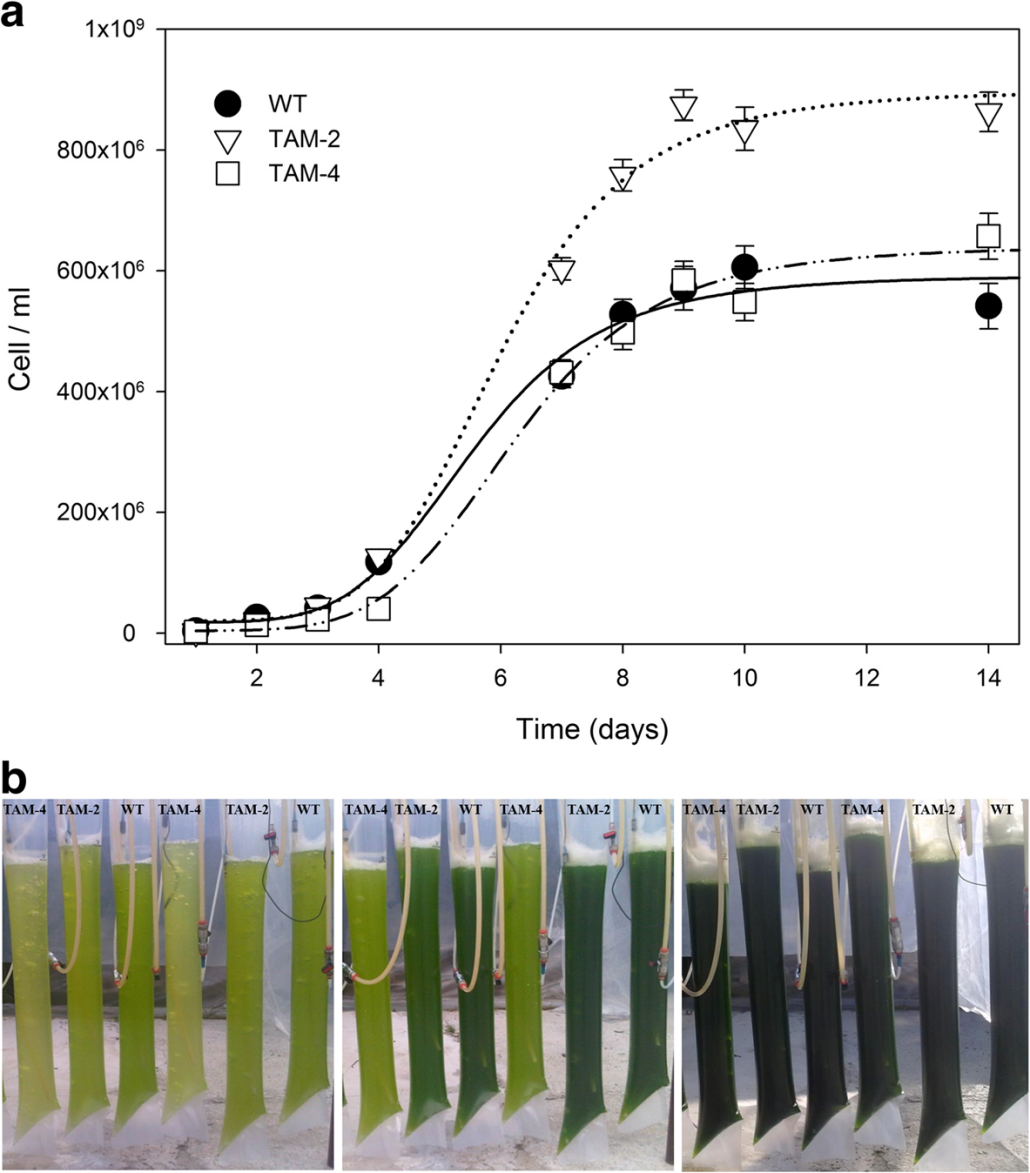
**Growth curves of wild-type, TAM-2, and TAM-4 mutants in outdoor photobioreactor. (a)** Time-dependent course of cell concentration. All experiments were performed in 7-L hanging bag reactors, fed with air/CO_2_ mix modulated in order to keep the pH of the medium always below 7.1. Two runs with nine reactors, each operated in parallel, were prepared. Data are expressed as mean ± SD, n =9. **(b)** Picture of the outdoor plant with 7-L reactors, showing growth stage of WT and TAM after two (left), four (center), and eight (right) days from initial inoculum.

The TAM-2 culture reached a cell concentration of about 8.3 · 10^8^ cell/ml at day six versus 5.2 · 10^8^ cell/ml in the WT (Figure 5a), with the specific growth rate (μ) for TAM-2 significantly higher than that for the WT (1.39 d^-1^ for TAM-2 and 1.28 d^-1^ for WT, Table 3). Moreover, the mutant showed a higher mean biomass productivity, equal to 380 mg per liter per day, that was significantly higher (+32%) than the corresponding value of 290 mg per liter per day for the WT (Table 3). In comparison, TAM-4 did not show any enhancement in either growth rate (1.30 d^-1^) or biomass productivity (300 mg per liter per day) over the WT (Figure 5b, Table 3). Further upscaling of the cultures was performed in outdoor conditions. To this aim, the three genotypes were cultivated in triplicate within 7-L hanging bags exposed to full natural light during September 2012. The temperature and light conditions, reported in Additional file 1: Figure S5, ranged between 15 and 22°C and between 500 and 2,200 μmol photons m^-2^ s^-1^. Figure 6 and Table 3 show that the final cellular concentration, the specific growth rate, and the daily biomass productivity were all significantly higher in the TAM-2 culture versus WT and TAM-4.

**Table 3:** **Growth parameters of wild-type, TAM-2, and TAM-4**, **cultured in air/CO**_**2**_ **bubbling systems**

	**Lab-scale, indoor PBR**		**Outdoor PBR**	
**Genotype**	**Mean increase of biomass (g L** ^**-1**^ **day** ^**-1**^ **)**	**μ (day** ^**-1**^ **)**	**Mean increase of biomass (g L** ^**-1**^ **day** ^**-1**^ **)**	**μ (day** ^**-1**^ **)**
WT	0.29 ± 0.01	1.28 ± 0.05	0.22 ± 0.02	1.25 ± 0.04
TAM-2	0.38 ± 0.01*	1.39 ± 0.03*	0.28 ± 0.03*	1.33 ± 0.04*
TAM-4	0.30 ± 0.02	1.30 ± 0.03	0.18 ± 0.03	1.25 ± 0.06

Biomass increase was measured in both lab-scale and outdoor photobioreactors, by the determination of dry biomass accumulated after the cultivation period, divided by the number of days of cultivation. μ, the specific growth rate, was calculated from the slope of the logarithmic cell concentration curve. Data are expressed as mean ± SD, n = 6. Significant different values (Student’s *t*-test, *P* <0.05) are marked with an asterisk.

## Discussion

The potential use of microalgal biomass for energy production has gained increasing attention in recent years, due to the many advantages over terrestrial crops [31]. However, algal biofuels are still more expensive than traditional fossil fuels because of a number of biological limitations [32]: among them is the inefficient conversion of solar energy into biomass under mass culture conditions. More than 80% of absorbed photons can thus be wasted at moderate to high irradiance, thus reducing photon use efficiencies and photosynthetic productivity [33] due to high optical density of the cell culture and the generation of a steep light gradient. Photosynthetic productivity can be improved with the design of new reactors with high surface-to-volume ratio [7] and the isolation of new strains with engineered optical properties.

In searching for mutants with improved optical properties we chose *Chlorella sorokiniana*, a robust species with a high market interest that offers the advantage of being able to carry out productivity tests at an industrial level. Although a reliable DNA transformation method has recently been developed for *C. sorokiniana* (Barbi T, Hiegle N, and Purton S, unpublished data), the targeted manipulation of specific genes such as *TLA1* [34] or *ARSA1* ([35]) that are known to be associated with antenna size, is not yet feasible for this species. Instead, a forward-genetic approach was adopted that involved random mutagenesis and screening for an altered fluorescence phenotype. The maximal level of fluorescence upon a pulse of saturating light is positively related to the number of Chl active in energy transfer to the PSII and is thus indicative of the antenna size of the strain [27]. Of the six putative mutants recovered from a screen of about 3,000 colonies, TAM-2 and TAM-4 showed a significant defect in PSII antenna size, as confirmed by the higher Chl *a*/*b* ratio. None of the six TAM mutants showed a reduced PSI antenna as expected, since the screening method using fluorescence induction at room temperature would not be selective for PSI antenna mutants.

The reduction in Chl content was similar in TAM-2 and TAM-4 (a reduction of about 39% with respect to WT Chl content/cell), and in both strains, the reduction was coupled to a modulation in the composition of the pigment-binding complexes with LHCII decreased to a similar extent (-49% in TAM-2, -38% in TAM-4). In contrast to the LHCII reduction, the amount of PSII per cell was found to be at WT levels, whereas the content of PSI was severely reduced, and to the same extent in both mutant strains (-34% compared to WT). Therefore, the two mutants, in addition to a reduced LHCII antenna content, also have a reduced content in PSI per cell, resulting in a photosynthetic phenotype resembling that of chloroplasts acclimated to excess light conditions [36].

Previous studies of *Chlamydomonas reinhardtii* chlorophyll-deficient mutants showed that a pale green phenotype could be due to different reasons rather than mutation affecting Lhcb-encoding genes, namely: mutations resulting in constitutive activation of NAB1, a repressor of the Lhc translation system in the cytoplasm [37], impairment in the mechanisms of protein import into the chloroplast [35] or Lhcb insertion in the thylakoids [27], mutations in Chl [35,38], or carotenoid biosynthesis pathways [39].

The observation that all six TAM mutants are resistant to high light (Figures 4 and 5) suggests that rather than defects in pigment biosynthesis, where lesions induce photosensitivity [40,41], mutations in the TAM lines likely affect chloroplast biogenesis steps such as those mediated by NAB1, ARSA1, or cpSRP [35,37,42]. Future work on the molecular analysis of such genes in the TAM mutants and complementation with the WT gene will confirm the causal link between antenna size and photosynthetic performance, and provide further insight into the basis of the phenotype. Furthermore, we cannot exclude that the lower PSI/PSII ratio derives from concomitant mutation(s) affecting their biosynthesis independently, but it is more likely that reduction of the PSI-LHCI complex is a secondary effect of PQ oxidation due to a decrease in PSII abundance/decrease in antenna size [43], as suggested from the unaffected stoichiometry between PSI and LHCI moieties of the complex (Figure 3b).

The measures of fluorescence induction in cells infiltrated with DCMU confirmed that, among the mutants, TAM-2 and TAM-4 have a marked reduction of the PSII antenna size as compared to WT, while PSI antenna size was unaffected.

Physiological characterization of WT and TAM-2 showed that photosynthetic yield was significantly enhanced in TAM-2. The linear phase of the light-saturation curve of O_2_ yield (between 0 and 300 μmol photons m^-2^ s^1^) showed the same slope, namely a similar quantum yield of photosynthesis in both strains under limiting light conditions, indicating that the decrease of antenna content and the differential reduction of the two photosystems did not negatively affect the efficiency of the photosynthetic apparatus. However, the P_max_ in TAM-2 was about 40% larger than in WT at saturating irradiances, indicating a higher productivity in high light. These results were consistent with the report that the *tla1* mutant of *C. reinhardtii* with a truncated antenna size has enhanced P_max_ [27]. Also, the *tla3* mutant showed enhanced photosynthetic productivity at saturating irradiances and a 60% reduction in the PSII functional antenna size caused by a defect in the insertion of chloroplast pre-proteins in thylakoids [42].

One might wonder if the depletion of antenna proteins and of photosystems could affect the photoautotrophic growth of strains. Under high irradiance, chloroplasts are particularly susceptible to photoinhibition [44], a phenomenon which impairs photosynthetic productivity. In higher plants, an extreme reduction in LHC proteins is obtained with the *ch1* mutation [45], in which assembly of LHC is prevented due to a lack of Chl *b*. Thylakoids isolated from *ch1* plants produce far more ^1^O_2_ with respect to that from WT plants, and are more sensitive to photo-oxidation, thus implying that functional LHC complexes are essential for photoprotection [18,46]. Since LHC antenna proteins have a central role in photoprotection, their depletion in the TAM mutants could have reduced the photochemical yield and increased photoinhibition. However, the light-saturation curve of photosynthesis showed no decline of oxygen evolution even at very high light intensity (5,000 μmol photons m^-2^ s^-1^). Moreover, the enhancement of photosynthetic yield by high light in TAM-2 with respect to WT suggests that phototolerance is not significantly affected. We conclude that a mutation that halves the LHC/PSII ratio and decreases the PSI content per cell does not result in photo-oxidative stress of *C. sorokiniana* cells under the growth conditions tested.

All the biochemical and spectroscopic analyses indicate that the TAM-2 is a good candidate for improved performance in a PBR system. To test this possibility, cell growth rates and biomass yield were measured in the long-term cultivation of dense algal suspensions under high irradiance. During seven days of growth TAM-2 showed a significant increase of productivity with respect to WT, both as biomass increment per day and maximal level of biomass reached at the end of the test period. The increased light use efficiency of TAM-2 was maintained upon further up-scaling of the PBR to 7 liters and 16 cm diameter exposed to full sunlight and natural temperature. These findings suggest that the improved productivity of such reduced antenna mutants is translatable from the lab to the industrial setting. Furthermore, it was assessed that the mutation procedure did not significantly affect the capacity of strains to undergo variable light/temperature conditions typical of outdoor biomass production.

A possible mechanism underlying the enhancement of biomass productivity is the thermal dissipation of ^1^Chl* in the bulk antenna, enhanced upon triggering the mechanisms of non-photochemical quenching (NPQ). A recent study on acclimation of *C. reinhardtii* to high light [47] showed that algal growth in high light unavoidably results in a reduction in the efficiency of light energy conversion into biomass, due to dissipation of a large fraction of photons absorbed as heat. Thus, unlike higher plants, in which the amplitude of NPQ is proportional to the actinic light intensity, algae dramatically upregulate their ability to perform NPQ even with moderate irradiances, thus leading to a strong reduction of photosynthetic efficiency [47]. The enhanced photosynthetic efficiency of TAM-2 suggested that this strain might be depleted in the heat dissipation response, thus showing an improved light energy conversion. Upon prolonged illumination of cells with the same intensity used for growth in lab-scale PBRs, the amplitude of NPQ at steady state was similar in WT and the TAM mutants. We conclude that the differential photosynthetic light use efficiency of WT versus TAM-2 was not due to differences in NPQ activity. It must be noted, however, that the NPQ amplitude observed with *Chlorella* was far below the level reported in *Chlamydomonas* [47], thus making any effect of NPQ level on productivity of limited impact.

From the characterization of TAM-2 strain we conclude that selection of strains by the reduction of optical cell density is a viable strategy to improve the light diffusion properties of the mass culture and to yield a higher productivity.

One question, however, is why reduction of both Chl content per cell and PSII antenna size did not improve photosynthetic productivity in TAM-4. In fact, the mutant showed a productivity far lower than TAM-2*,* consistent with the low P_max_ value measured, and yet a reduced LHC/PSII was observed as in TAM-2. One hypothesis is that imbalance in the PSII/PSI ratio could affect the photosynthetic electron transport rate [48]. However, the PSII/PSI ratio was increased by the same extent in both the TAM-2 and TAM-4 mutants, suggesting that the differential growth phenotype of TAM-2 versus TAM-4 is unlikely to be due to limitations in PSI and PSII electron transport rate. Since TAM-2 and TAM-4 have a similar Chl content per cell and PSII antenna size, their different performance implies that reducing antenna size and Chl per cell is not enough to obtain a better light use efficiency in dense cultures. This is consistent with results by [49] reporting on two low Chl/cell mutants of *Cyclotella sp*. which did not gain in productivity with respect to WT when grown in semi-continuous, laboratory-scale PBRs. A possible explanation can be proposed on the basis of the increased respiration rate of TAM-4 with respect to TAM-2. Mutants generated by random UV or chemical mutagenesis are likely to induce multiple mutations in any single cell. Some of these mutations could negatively affect the metabolic network of the cell, thus causing slower growth. A lower LHC content *per se* had no consequences on the respiration capacity of the cells [27]. Thus, an increased respiration rate in TAM-4 cells can be attributed to additional mutations, which adversely impact cell metabolism. Therefore, the distinctive parameter that needs to be assessed, beyond lower Chl content per cell and reduced PSII antenna size, is the P_max_/respiration rate, which indeed was not significantly affected in TAM-2 cells, but was reduced by more than 50% in TAM-4. Thus, a full photosynthetic characterization of mutants obtained by chemical/UV mutagenesis is needed to ensure that mutants with a truncated antenna are not affected in their photosynthetic performance in ways other than reduced LHC content. The concept is consistent with that reported by the researchers of [27], who performed a screening of over 6,000 *C. reinhardtii* colonies from a library obtained through DNA insertional mutagenesis. Although the initial screen resulted in 129 putative truncated antenna mutants, only one showed an improvement in photosynthetic efficiency.

Further molecular characterization of the TAM mutants would allow the identification of genes which modulate the LHC content of algal cells. Indeed, new genome sequencing technologies provide an opportunity to identify such sites of mutation [50], and desired traits might then be transferred to other species of interest to the algal biotechnology industry, for CO_2_ mitigation processes or production of biomass, biofuels, or high-value products [32,51-54].

## Conclusions

Our results show that the modulation of antenna size to improve light penetration and enhance photosynthetic yield is a promising strategy in the development of domesticated microalgal strains for mass culture. However, it is important to consider that the optical path length of our growth facilities was short and likely to be significantly increased in industrial-scale outdoor PBRs, thus increasing the light gradient effect and further favoring the growth rate of TAM-2 with respect to WT. Furthermore, with this round of mutagenesis we selected only six lines whose maximal reduction in LHC antenna proteins was 40% with respect to WT. Calculations suggest that a maximal P_max_ could be achieved in algal strains with Chl content/cell below 20% [22]; thus, even “paler” mutants than TAM-2 could further increase the photosynthetic performance in mass culture.

## Methods

### Cell cultivation

*Chlorella sorokiniana* 7-11-05 [55] was obtained from the UTEX Culture Collection (University of Texas, Austin, TX [http://web.biosci.utexas.edu/utex/]) as strain UTEX1230; maintained on BG-11 agar plates [56] and grown photoautotrophically in BG-11 medium in flasks at 25°C, 70 μmol photons m^-2^ s^-1^, with a photoperiod of 16:8 h light:dark. Irradiance was provided by warm-white fluorescent lamps. For physiological measurements, cultures were harvested during the logarithmic growth phase (about 1-3 · 10^7^ cells ml^-1^).

### Isolation of mutants

10 ml of a mid-log culture (about 10^8^ cells) was subjected to UV irradiation using a 6-W UV bulb (254 nm), under predetermined conditions that yielded a 10% survival rate. The cells were left to recover in the dark for 2 h to prevent photoreactivation followed by plating at 100-fold dilution on acetate-containing (TAP) medium [57]. Single colonies appeared after seven days and approximately 3,000 were tooth-picked to fresh TAP medium. Mutants with low Chl fluorescence were identified by spotting cultures of each onto both TAP and minimal media; allowing growth in the light for seven days; dark-adapting for 10 min, and directly measuring *in vivo* fluorescence using a FluorCam 700MF (Photon Systems Instruments, Brno, Czech Republic). The F_max_ value was recorded following an 800-ms flash of saturating white light and displayed as a false-color image for each spot [27]. Strains still capable of phototrophic growth but showing significantly reduced fluorescence were retained for further analysis.

### Cell count and pigment analysis

Cell density was measured using an improved Neubauer hemocytometer. Pigments were extracted from thylakoids with 85% acetone buffered with Na_2_CO_3_ or from intact cells with dimethylformamide. The supernatant of each sample was recovered after centrifugation (10 min at 15,000 g, 4°C); then the pigments were separated and quantified by HPLC [58].

### Measurements of photosynthetic activity

The oxygen evolution activity of the cultures was measured at 25°C with a Clark-type O_2_ electrode (Hansatech, Norfolk, UK), upon illumination with light from a halogen lamp (Schott, Germany). Samples of 2-ml cell suspension, 5 · 10^6^ cell ml^-1^, were loaded into the oxygen electrode chamber. NaHCO_3_ (3 mM final concentration) was added to the cell suspension prior to the O_2_ evolution measurements to ensure that electron transport was not limited by the carbon supply.

### Isolation of thylakoid membranes

The cells were harvested by centrifugation at 1,500 g for 5 min at 4°C. The samples were resuspended with ice-cold grinding buffer (0.35 M sorbitol, 50 mM Tricine pH 7.9, 10 mM NaCl, 5 mM MgCl_2_, 0.5% fat-free milk powder, 0.5 ml l^-1^ Antifoam A silicon polymer (Sigma), 1 mM aminocaproic acid, 1 mM aminobenzamidine, and 100 mM phenylmethylsulfonyl fluoride) at a final concentration of 10^8^ cell ml^-1^, and disrupted by passing three times through an ice-cold cell disruptor (Constant Systems, Northants, UK) at 1.48 kbar. The homogenate was then centrifuged at 1,500 g for 5 min, and the supernatant was further centrifuged at 30,000 g for 20 min at 4°C. The thylakoid membrane pellet was resuspended in a buffer containing 50% (w/v) glycerol, 20 mM Tricine pH 7.9, 10 mM NaCl, 5 mM MgCl_2_, 1 mM aminocaproic acid, 1 mM aminobenzamidine, and 100 mM phenylmethylsulfonyl fluoride, and immediately used for analysis, or frozen in liquid nitrogen and stored at -80°C.

### Gel electrophoresis and immunoblotting

SDS-PAGE analysis was performed with the Tris-Tricine buffer system [59]. Non-denaturing Deriphat-PAGE was performed following the method developed in [60] with the modification described in [36]. Thylakoids concentrated at 1 mg/ml Chls were solubilized with a final 1% β-DM, and 20 μg of Chls were loaded in each lane. For immunotitration, thylakoid samples corresponding to 0.25, 0.5, 0.75, and 1.0 μg of Chls were loaded for each sample and electroblotted on nitrocellulose membranes. Proteins were detected with alkaline phosphatase-conjugated antibody, according to [61]. The signal amplitude was quantified (n =3) using GelPro 3.2 software (Bio-Rad). In order to avoid any deviation between different immunoblots, samples were compared only when loaded on the same gel.

### *In vivo* Chl fluorescence analysis

Fluorescence induction kinetics were recorded with a home-built apparatus, previously described [62]. For measurements of PSII functional antenna size, variable fluorescence was induced with a green light of 15 μmol photons m^-2^ s^-1^ on dark-adapted cells (about 1.0 · 10^7^ cells/ml) in BG-11 medium supplemented with 50 μM DCMU. The F_0_ values were subtracted from each curve, and the fluorescence inductions were normalized to the same F_v_ in order to estimate antenna size more accurately. The reciprocal of time corresponding to two-thirds of the fluorescence rise (T_2/3_) was taken as a measure of the PSII functional antenna size [30]. PSII function during photosynthesis was measured through Chl fluorescence on cell suspensions at room temperature with a PAM 101 fluorimeter (Heinz-Walz, Effeltrich, Germany), with a saturating light pulse of 4500 μmol photons m^-2^ s^-1^, 0.6 s, and white actinic light of 500 μmol photons m^-2^ s^-1^, supplied by a KL1500 halogen lamp (Schott). The NPQ values were calculated according to [63].

### Analysis of P700 redox state

Spectroscopic measurements were performed on the thylakoids using an LED spectrophotometer (JTS-10, Bio-Logic Science Instruments, France) in which absorption changes are sampled by weak monochromatic flashes (10-nm bandwidth) provided by light emitting diodes (LEDs). The relative antenna size of PSI was determined by analyzing time courses of P700 photo-oxidation upon illumination of the thylakoid suspension with weak far-red light (12 μmol photons m^-2^ s^-1^). The reaction mixture contained 20 mM Tricine pH 7.9, 10 mM NaCl, 5 mM MgCl_2_, 50 μM 2,5-dibromo-3-methyl-6-isopropylbenzoquinone (DBMIB), 1 mM methylviologen, and thylakoid membranes corresponding to 75 μg Chls ml^−1^.

### Growth analysis/indoor PBR

The indoor growth experiments were performed at 25°C in home-built indoor photobioreactors (PBRs), composed of glass cylinders with a maximum light path of 8 cm and a working volume of 1 L each. The cultures were continuously mixed with a mixture composed of air and CO_2_. The ratio of compressed air and CO_2_ was automatically adjusted to keep the pH of the medium below 7.1. Each autotrophic batch cultivation was carried out in duplicate. The medium, cylinders and tubes were sterilized in an autoclave for 20 min at 121°C in order to prevent any contamination. Illumination was provided by a panel of warm-white LEDs, and the microalgae were exposed to an irradiance of 450 μmol photons m^-2^ s^-1^ with a photoperiod of 16:8 h light:dark. The parameters determined to monitor cell growth were cell number and dry biomass weight, for which the washed cell pellets were dried overnight in a lyophilizer.

### Growth analysis/outdoor PBR

The outdoor growth experiments were performed in a PBR located in Sommacampagna (Verona, Italy) during September 2012. The plant consisted of nine cylindrical hanging bags with a diameter of around 16 cm and a reactor volume of 7 L. The gas flow rate was supplied through a perforated plastic hose at the bottom of the reactor, which allowed efficient intermixing of the cell suspension. The CO_2_ flow rate was continuously adjusted by a controller unit and added to the air flow in order to maintain the pH of the medium below 7.1. The average light intensity and temperature measured during the course of the experiment are displayed in Additional file 1: Figure S5.

## Additional file

Additional file 1: Figure S1. Schematic illustration of the mutagenesis and screening strategy used to isolate antenna mutants. **Figure S2.** Functional antenna size of PSII (A, B) and PSI (C, D) measured in wild-type and TAM mutants. See methods for details.. Data are expressed as mean ± SD, n =7. **Figure S3.** Light-saturation curves of photosynthesis. Curves were obtained with the *C. sorokiniana* wild-type and TAM mutants. Data are expressed as mean ± SD, n =4. **Figure S4.** Kinetics of formation and relaxation of photoprotective energy dissipation in wild-type and TAM mutants. See methods for details. Symbols and error bars show means ± SD (n =3). **Figure S5.** Daily mean irradiance on the reactors’ surface (left panel) and atmospheric temperature (right panel), measured during the outdoor experiments. **Table S1.** Pigment composition of wild-type and TAM mutants. Data are expressed as mean ± SD. Significantly different values (ANOVA, *P* <0.05) with respect to the wild type, within the same column, are marked with different letters. **Table S2.** HPLC analysis of carotenoid composition of wild-type and TAM mutants. Data are expressed as mean ± SD and normalized to 100 Chls. Significantly different values (ANOVA, *P* <0.05) with respect to the wild type, within the same column, are marked with different letters. **Table S3.** Relative abundance of pigment-protein complex in the wild-type and TAM mutants. Amount of pigment-protein complexes per cell were calculated by densitometric analysis of native PAGE and by Chls content per cell, and expressed as a percentage of the corresponding wild-type values. Data are expressed as means ± standard deviation (n =3). Significantly different values (ANOVA, *P* <0.05) with respect to the wild type, within the same column, are marked with different letters.

## Abbreviations

β-DM: dodecyl-β-D-maltoside
^1^Chl*: singlet excited state of Chl
Chl: chlorophylls
DCMU: 3-(3,4-dichlorophenyl)-1,1-dimethylurea
DBMIB: 2,5-dibromo-3-methyl-6-isopropylbenzoquinone
DW: dry weight
LHCI/II: light harvesting complex of PSI/II
NPQ: non-photochemical quenching
PBR: photobioreactor
PQ: plastoquinone
PSI/II: photosystem I/II
P700: reaction center of PSI
ROS: reactive oxygen species
TAM: truncated antenna mutant
WT: wild-type
