# Supplementary material for: Domestication of the green alga Chlorella sorokiniana: reduction of antenna size improves light-use efficiency in a photobioreactor
Source: Biotechnol Biofuels. 2014 Oct 21;7:157. doi: 10.1186/s13068-014-0157-z (PMC4210543; doi:10.1186/s13068-014-0157-z)
Supplement: Additional file 1: Figure S1. — Schematic illustration of the mutagenesis and screening strategy used to isolate antenna mutants. Figure S2. Functional antenna size of PSII (A, B) and PSI (C, D) measured in wild-type and TAM mutants. See methods for details.. Data are expressed as mean ± SD, n =7. Figure S3. Light-saturation curves of photosynthesis. Curves were obtained with the C. sorokiniana wild-type and TAM mutants. Data are expressed as mean ± SD, n =4. Figure S4. Kinetics of formation and relaxation of photoprotective energy dissipation in wild-type and TAM mutants. See methods for details. Symbols and error bars show means ± SD (n =3). Figure S5. Daily mean irradiance on the reactors’ surface (left panel) and atmospheric temperature (right panel), measured during the outdoor experiments. Table S1. Pigment composition of wild-type and TAM mutants. Data are expressed as mean ± SD. Significantly different values (ANOVA, P <0.05) with respect to the wild type, within the same column, are marked with different letters. Table S2. HPLC analysis of carotenoid composition of wild-type and TAM mutants. Data are expressed as mean ± SD and normalized to 100 Chls. Significantly different values (ANOVA, P <0.05) with respect to the wild type, within the same column, are marked with different letters. Table S3. Relative abundance of pigment-protein complex in the wild-type and TAM mutants. Amount of pigment-protein complexes per cell were calculated by densitometric analysis of native PAGE and by Chls content per cell, and expressed as a percentage of the corresponding wild-type values. Data are expressed as means ± standard deviation (n =3). Significantly different values (ANOVA, P <0.05) with respect to the wild type, within the same column, are marked with different letters. [file 13068_2014_157_MOESM1_ESM.pdf]

Supporting information

Figure S1. Schematic illustration of the mutagenesis and screening strategy used to isolate antenna mutants.

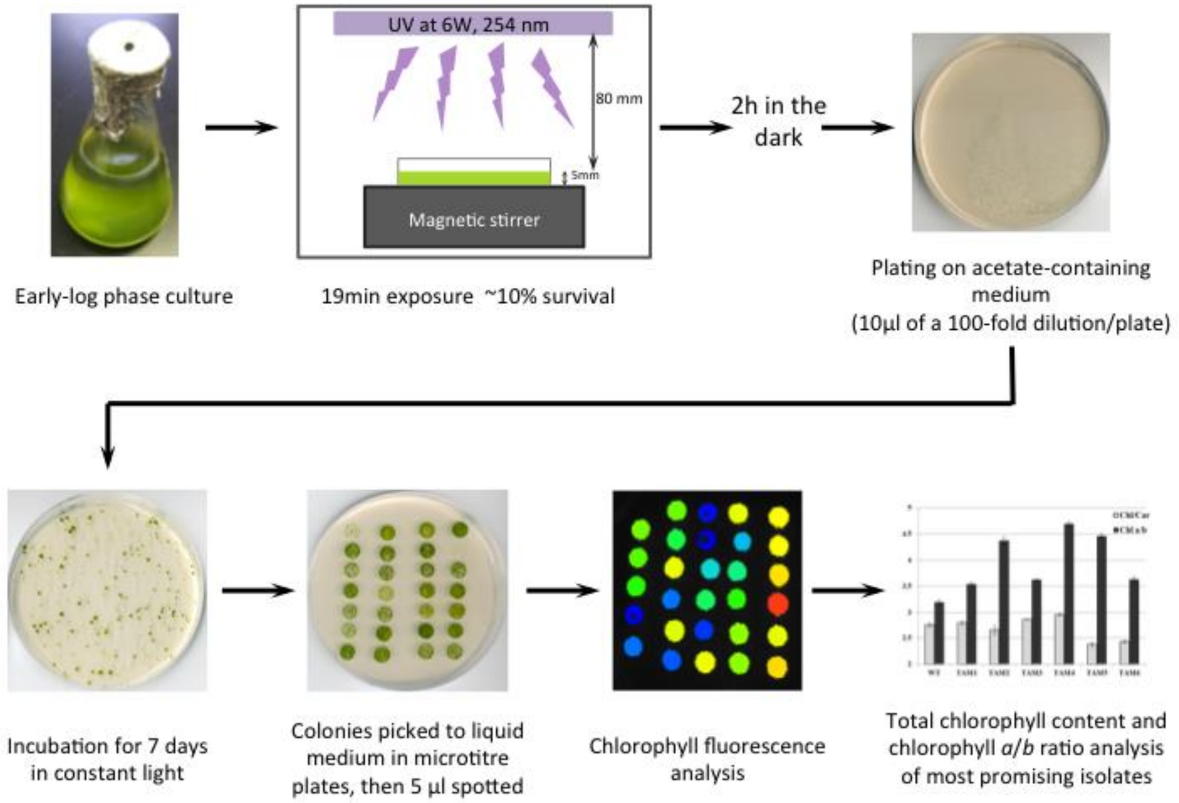

Figure S2. Functional antenna size of PSII and PSI measured in wild-type and TAM mutants (A) Variable Chl fluorescence was induced with a green light of 15 µmol photons m^{-2} s^{-1}, on dark-adapted cells (~$1.0 \cdot 10^7$ cells/ml) in BG-11 medium supplemented with 50 µM DCMU. The reciprocal of time corresponding to two-thirds of the fluorescence rise ($T_{2/3}$) was taken as a measure of the PSII functional antenna size. (B) The kinetics of P700 oxidation (ΔAbs at 705 nm) were measured on thylakoids suspension (75 µg Chl/ml) treated with 50 µM DBMIB and 1 mM methylviologen, upon illumination with a 10 s pulse of red actinic light (λ = 630 nm, 560 µmol photons m^{-2} s^{-1}). Data are expressed as mean ± SD, n = 7.

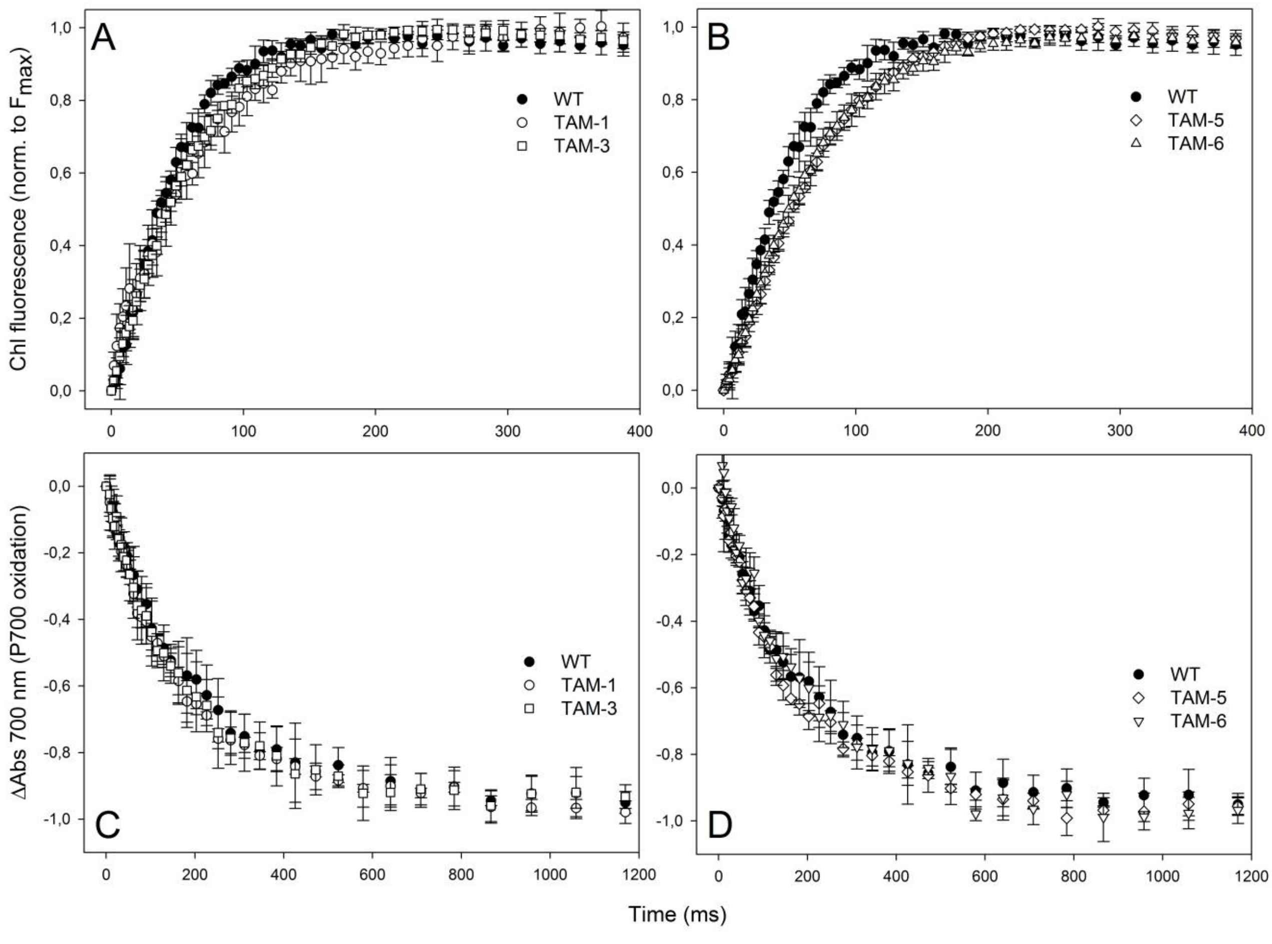

Figure S3. Light-saturation curves of photosynthesis. Curves were obtained with the *C. sorokiniana* wild-type and TAM mutants. Data are expressed as mean ± SD, n = 4.

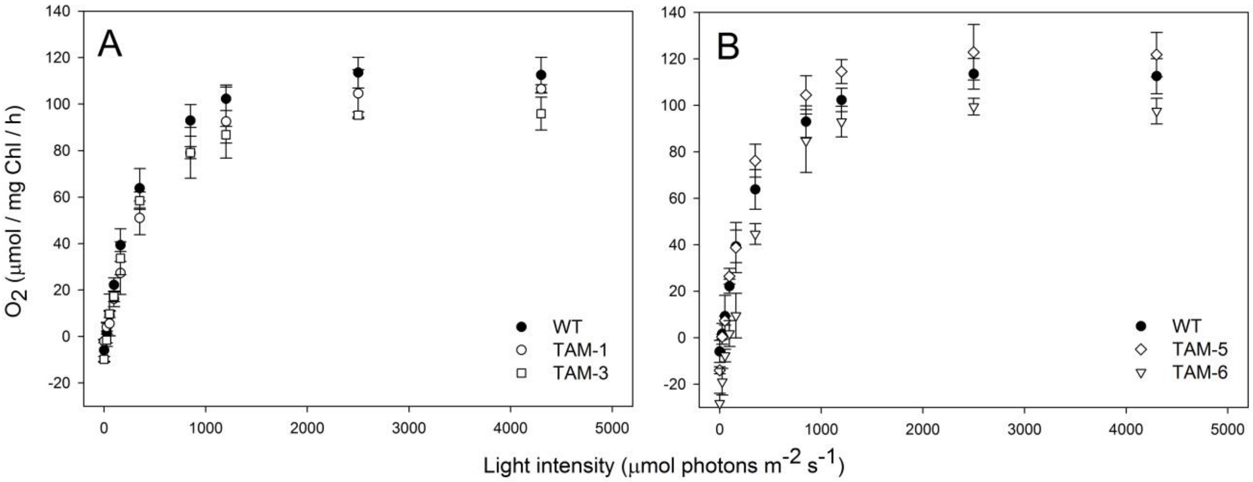

Figure S4. Kinetics of formation and relaxation of photoprotective energy dissipation in wild-type and TAM mutants. NPQ kinetics were measured on wild-type, TAM-2 and TAM-4 cells, grown photoautotrophically, upon illumination with 500 µmol photons m^{-2} s^{-1} of white actinic light. Symbols and error bars show means ± standard deviation (n = 3).

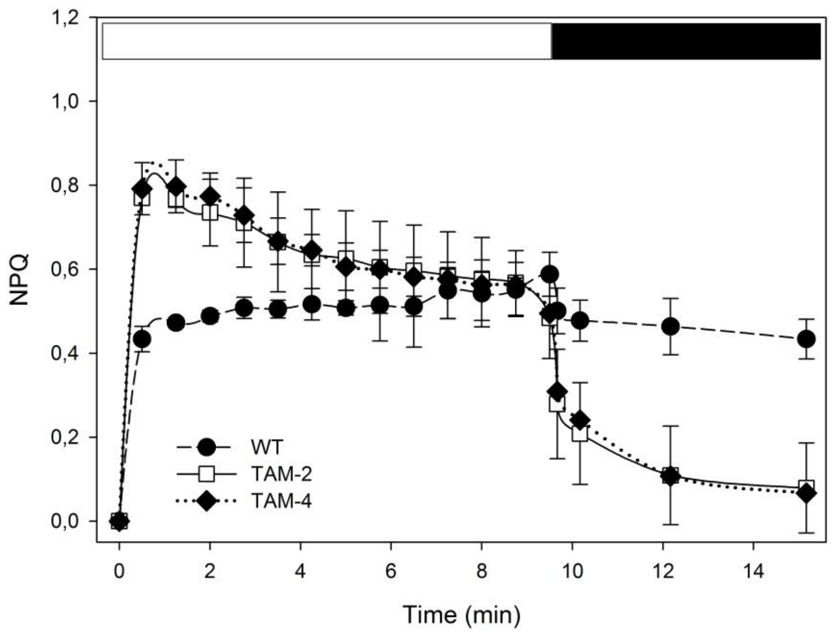

Figure S5

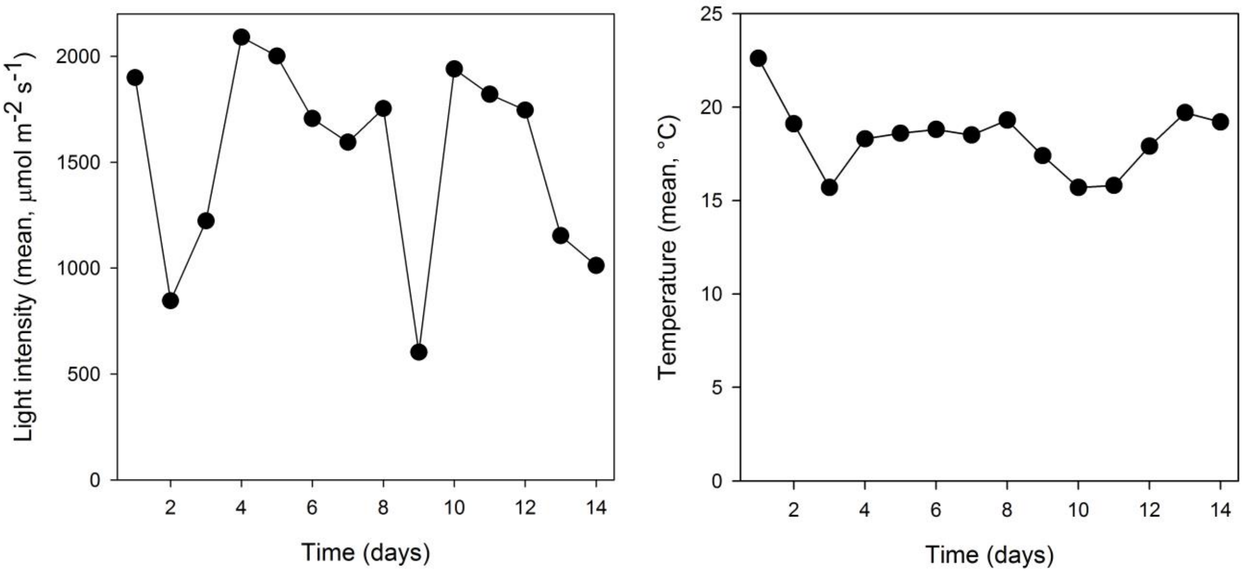

Figure S5. Daily mean irradiance on the reactors' surface (left panel) and mean atmospheric temperature (right panel) measured during the outdoor experiment.

Table S1. Pigment composition of wild-type and TAM mutants. Data are expressed as mean ± SD. Significantly different values (ANOVA, $P < 0.05$) with respect to the wild-type, within the same column, are marked with different letters.

genotype	Chl / cell (pg)	Chl *a / b*	Chl / Car	PSII antenna size ($T_{2/3}^{-1} \cdot 10^3$, ms^{-1})
**WT**	0.49 ± 0.07[a]	2.62 ± 0.02[a]	3.43 ± 0.02[a]	18.2 ± 1.4[a]
**TAM-1**	0.50 ± 0.09[a]	2.92 ± 0.03[b]	3.16 ± 0.02[b,c]	14.5 ± 1.6[b]
**TAM-2**	0.30 ± 0.02[b]	3.36 ± 0.02[c]	3.07 ± 0.02[b]	10.5 ± 0.5[c]
**TAM-3**	0.58 ± 0.09[a]	2.69 ± 0.12[d]	3.47 ± 0.12[a]	15.5 ± 0.5[b]
**TAM-4**	0.34 ± 0.05[b]	3.41 ± 0.03[c]	3.18 ± 0.03[b,c]	9.4 ± 0.5[c]
**TAM-5**	0.52 ± 0.08[a]	2.84 ± 0.03[b]	3.30 ± 0.04[a,c]	12.9 ± 0.5[b]
**TAM-6**	0.51 ± 0.07[a]	2.91 ± 0.02[b]	3.35 ± 0.02[a]	12.9 ± 0.8[b]

Table S2. HPLC analysis of carotenoid composition of wild-type and TAM mutants. Cells were dark-adapted before pigment extraction in DMFA. Data are expressed as mean ± SD and normalized to 100 Chls. Significantly different values (ANOVA, $P < 0.05$) with respect to the wild-type, within the same column, are marked with different letters.

genotype	mol pigment / 100 mol Chls						
	neoxanthin	violaxanthin	antheraxanthin	lutein	zeaxanthin	α-carotene	β-carotene
**WT**	6.6 ± 0.2[a]	1.5 ± 0.1[a]	0.6 ± 0.1[a]	17.3 ± 0.3[a]	0.8 ± 0.1[a]	0.5 ± 0.2[a]	2.1 ± 0.2[a]
**TAM-1**	6.2 ± 0.2[a]	2.0 ± 0.1[a]	0.5 ± 0.1[a]	17.3 ± 0.1[a]	1.1 ± 0.1[a]	0.8 ± 0.2[a]	2.1 ± 0.1[a]
**TAM-2**	5.8 ± 0.2[b]	5.9 ± 0.1[b]	1.9 ± 0.1[b]	10.3 ± 0.2[b]	2.6 ± 0.2[b]	0.2 ± 0.1[a]	3.7 ± 0.3[b]
**TAM-3**	6.5 ± 0.1[a]	2.1 ± 0.1[a]	1.2 ± 0.1[c]	14.2 ± 0.3[c]	2.2 ± 0.1[b]	0.4 ± 0.2[a]	2.3 ± 0.1[a]
**TAM-4**	6.0 ± 0.1[b]	6.9 ± 0.1[c]	1.9 ± 0.1[b]	9.6 ± 0.1[d]	2.4 ± 0.2[b]	0.2 ± 0.1[a]	3.9 ± 0.2[b]
**TAM-5**	6.7 ± 0.3[a]	3.1 ± 0.1[d]	2.8 ± 0.1[d]	9.4 ± 0.2[d]	3.7 ± 0.3[c]	0.2 ± 0.1[a]	2.8 ± 0.2[c]
**TAM-6**	6.3 ± 0.2[a]	3.6 ± 0.1[d]	1.4 ± 0.1[c]	13.2 ± 0.3[c]	2.1 ± 0.1[b]	0.4 ± 0.1[a]	2.4 ± 0.2[a]

Table S3. Relative abundance of pigment-protein complex in the wild-type and TAM mutants. Amount of pigment-protein complexes per cell were calculated by densitometric analysis of native PAGE and by Chls content per cell, and expressed as a percentage of the corresponding wild-type values. Data are expressed as means ± standard deviation (n = 3). Significantly different values (ANOVA, $P < 0.05$) with respect to the wild-type, within the same column, are marked with different letters.

Genotype	Relative abundance of pigment-protein complexes per cell		
	**PSI-LHCI**	**PSII core**	**Lhcb**
**WT**	100 ± 8^{a}	100 ± 9^{a}	100 ± 7^{a}
**TAM-2**	66 ± 4^{b}	102 ± 4^{a}	51 ± 3^{b}
**TAM-4**	66 ± 11^{b}	107 ± 6^{a}	62 ± 6^{b}
